# Chilling and Drought Stresses in Crop Plants: Implications, Cross Talk, and Potential Management Opportunities

**DOI:** 10.3389/fpls.2018.00393

**Published:** 2018-04-10

**Authors:** Hafiz A. Hussain, Saddam Hussain, Abdul Khaliq, Umair Ashraf, Shakeel A. Anjum, Shengnan Men, Longchang Wang

**Affiliations:** ^1^Key Laboratory of Eco-Environments in Three Gorges Reservoir Region, Engineering Research Center of South Upland Agriculture, Ministry of Education, College of Agronomy and Biotechnology, Southwest University, Chongqing, China; ^2^Department of Agronomy, University of Agriculture Faisalabad, Faisalabad, Pakistan

**Keywords:** climate change, chilling, drought, plant responses, stress management

## Abstract

Plants face a combination of different abiotic stresses under field conditions which are lethal to plant growth and production. Simultaneous occurrence of chilling and drought stresses in plants due to the drastic and rapid global climate changes, can alter the morphological, physiological and molecular responses. Both these stresses adversely affect the plant growth and yields due to physical damages, physiological and biochemical disruptions, and molecular changes. In general, the co-occurrence of chilling and drought combination is even worse for crop production rather than an individual stress condition. Plants attain various common and different physiological and molecular protective approaches for tolerance under chilling and drought stresses. Nevertheless, plant responses to a combination of chilling and drought stresses are unique from those to individual stress. In the present review, we summarized the recent evidence on plant responses to chilling and drought stresses on shared as well as unique basis and tried to find a common thread potentially underlying these responses. We addressed the possible cross talk between plant responses to these stresses and discussed the potential management strategies for regulating the mechanisms of plant tolerance to drought and/or chilling stresses. To date, various novel approaches have been tested in minimizing the negative effects of combine stresses. Despite of the main improvements there is still a big room for improvement in combination of drought and chilling tolerance. Thus, future researches particularly using biotechnological and molecular approaches should be carried out to develop genetically engineered plants with enhanced tolerance against these stress factors.

## Introduction

Crops grown under open environments often pass through periods of abiotic stress during their life cycle. Such stresses many a time overlap so that the crop growth and productivity of the crops is adversely affected. Plants pass through a series of morphological, physiological, biochemical, and molecular changes in a quest to mitigate such adversities of the abiotic stresses. A lot of work reports the adaptive responses of crops plants to different abiotic stresses wherein emphasis has been laid on individual stress factors (e.g., Jongdee et al., 2002; Chinnusamy et al., 2007; Nejad et al., 2010; Basu et al., 2016; Wang et al., 2016; Anjum et al., 2017). Nonetheless, understanding the plant responses to combined stress factors is inevitable for enhancing the adaptation of plants under field conditions (Pandey et al., 2015). Crops experience periods of extreme low temperatures in many regions of the world (Ruelland et al., 2009; Wang et al., 2016), and are exposed to limited water availability owing to either drought or disturbed water movement and uptake under low ambient temperatures (Shinozaki et al., 2003; Zhang J.Z. et al., 2004; Beck et al., 2007). Such an exposure of plants to chilling and drought simultaneously hampers plant growth, and hence is detrimental to productivity (Tommasini et al., 2008). Studies show that primarily chilling and drought pose a similar impact on stomatal development and leaf growth, nevertheless, the mechanisms of drought-induced changes in some physiological processes are quite different than those induced by chilling (Deng et al., 2012). A little is known about the combined effect of chilling and drought stresses on plants, and whether or not plant responses to these are unique or shared is also unclear. Plants may exhibit common molecular and physiological responses on exposure chilling and drought (shared response), others may be specific to a given stress factor (Atkinson et al., 2013; Sewelam et al., 2014). In general, chilling stress thermodynamically declines the kinetics of several physiological as well as metabolic processes occurring in plants (Ruelland et al., 2009; Hussain et al., 2016). It severely reduces the rate and uniformity of germination, hampers seedling vigor, and delays ontogenic plant development (Cruz and Milach, 2004; Oliver et al., 2007), resulting in severe crop yield losses (Ruelland et al., 2009).

Drought stress in plants is characterized by reduced leaf water potential and turgor pressure, stomatal closure, and decreased cell growth and enlargement (Farooq et al., 2009b). Drought stress reduces the plant growth by influencing various physiological as well as biochemical functions such as photosynthesis, chlorophyll synthesis, nutrient metabolism, ion uptake and translocation, respiration, and carbohydrates metabolism (Jaleel et al., 2008; Farooq et al., 2009b; Li et al., 2011). Nevertheless, plants experience water deficit not only during drought, but low temperature may also cause turgor stress at cellular level (Thomashow, 1994; Janska et al., 2009; Yadav, 2010) owing to poor root hydraulic conductance and diminished root activity (Aroca et al., 2003).

Oxidative stress is crucial in relation to chilling- and drought-induced injuries in plants (Srivalli et al., 2003; Hussain et al., 2016). Drought and chilling stress exacerbates ROS production in plants’ cell. Excess production and the accumulation of ROS causes oxidative damage at cellular level, disrupts cellular membranes, and leads to enzyme inactivation, protein degradation, and ionic imbalance in plants (Baier et al., 2005; Tarchoune et al., 2010). The ROS disturb the cellular macromolecules including DNA, and hence may cause deletion of bases due to alkylation and oxidation which are linked with various physiological and biochemical disorders in plants (Tuteja and Tuteja, 2001). Plants possess a highly efficient and sophisticated antioxidative defense system to control the overproduction of ROS (Hussain et al., 2016). The ROS-induced damages and disruption of cellular homeostasis are alleviated by the action of different enzymatic (e.g., catalase, CAT; superoxide dismutase, SOD; peroxidase, POD; glutathione reductase, GR; glutathione peroxidase, GPX) and non-enzymatic (e.g., ascorbic acid, carotenoids, α-tocopherol, and glutathione content) antioxidants (Gill and Tuteja, 2010; Chen et al., 2016; Hussain et al., 2016). In plants, the levels of ROS are regulated by their production rate as well as the extent of their detoxification by enzymatic and/or non-enzymatic antioxidant systems. Mechanism of ROS production and its scavenging by high antioxidative capacity has been associated with tolerance of plants to abiotic stresses (Gill and Tuteja, 2010).

Studies involving chilling and drought as absolute stress factor have been well documented for several crops. Nevertheless, little focus has been laid on their combined effect, and plant response to both of these. Some recent studies ponder that the physiological and molecular response of the crops to combination of two different environmental stresses is unique so that such responses cannot be directly extrapolated when looked in the context of any of these stress applied individually (Mittler, 2006). Based on the available work on the effect of multiple stresses on crop plants, an effort is made to comprehend the current understanding of chilling and drought stress on crop plants. We have tried to give a general overview of shared and unique responses of crop plants to drought and chilling stresses and discuss some possible mitigation strategies to cope with these stresses for minimizing their damage on crop plants.

## Plant Responses to Chilling and Drought Stresses

Water scarcity and suboptimal temperature at any growth stage of crop plants evoke negative effects on crop growth and development. Such detrimental effects on plants depend on the magnitude of stress and its period and the plant growth stage. Such effects are elicited in terms of morphological, physiological, biochemical, and molecular processes in plants.

## Morphological and Yield Responses

### Germination

Some field crops are extremely sensitive to chilling and drought particularly during germination and early phases of seedling development. Each seed requires optimum temperature and soil moisture for germination. Chilling stress severely impairs the germination and often lowers the seedling vigor (Kang and Saltveit, 2002; Wang et al., 2016), delays plant development and ultimately causes severe yield losses (Cruz and Milach, 2004; Oliver et al., 2007; Ruelland et al., 2009). Chilling stress is known to thermodynamically limit the kinetics of various physiological as well as metabolic functions in plants (Ruelland et al., 2009). Chilling stress evokes shoot water deficit due to its thermophilic nature and reduced root water uptake (Stewart et al., 1990).

Drought stress impairs germination by limiting water imbibition, and reduces seedling vigor (Kaya et al., 2006; Farooq et al., 2009b). Both chilling stress and limited water supply results in disturbance of osmotic balance, impaired metabolic activity at cellular level and excessive ROS production leading to alterations in DNA, RNA and protein structures, membrane damage, reduced respiration and less ATP production (Priestley, 1986) ultimately causing loss in seed germination and vigor.

### Growth

Both chilling and drought stress are known to cause significant reductions in plant growth and development. Various studies have reported the negative effects of chilling stress on growth of rice (Hussain et al., 2016; Wang et al., 2016), cotton (Zhao et al., 2012), tomato (Starck et al., 2000), potato (Svensson et al., 2002), muskmelons (Wang et al., 2004), and sugarcane (Thakur et al., 2010; Anjum et al., 2011; Zhu et al., 2013). Likewise, drought stress has also been reported to reduce plant height, leaf area, stem diameter, and plant biomass in different field crops (Apel and Hirt, 2004; Farooq et al., 2009b; Zheng et al., 2016). Our recent investigation on maize (Hussain et al., unpublished) has revealed that chilling as well as drought stress severely damaged the shoot and root growth attributes, compared with control. The interactive damage caused by chilling and drought was found to be additive for all growth attributes.

Chilling stress may cause necrotic lesions on leaves, delay leaf development, prolong cell cycle with decreased cell production, induce wilting, and increase susceptibility to pathogens and diseases (Korkmaz and Dufault, 2001; Rymen et al., 2007). Decreased rate of both cell division and elongation causes smaller leaf area (Ben-Haj-Salah and Tardieu, 1995; Jouyban et al., 2013), while slow leaf initiation rate leads to reduced number of leaves under periods of chilling stress (Warrington and Kanemasu, 1983; Lukatkin et al., 2012; Jouyban et al., 2013). Under drought stress, leaf growth is mainly restricted by decreased leaf water potential (Hsiao and Xu, 2000). Whilst, interrupted water flow from xylem to other elongating cells and reduced turgor pressure under severe drought stress, leads to reduced cell elongation and smaller leaf area in crop plants (Nonami, 1998; Schuppler et al., 1998; Taiz and Zeiger, 2006).

Chilling stress restricts root growth by decreasing root length, biomass, and morphology (Cutforth et al., 1986), which ultimately reduces volume of the root system for exploring the nutrients and water (Richner et al., 1996). Nevertheless, the reported effects of drought stress on root growth have been diverse. For instance, drought stress increased the root length of *Catharanthus roseus* (Jaleel et al., 2008) and sunflower (Tahir et al., 2002), but did not affect the root growth of wheat and maize (Sacks et al., 1997). Increase in root length under drought conditions have been attributed to increased abscisic acid content in roots (Manivannan et al., 2007). Wullschleger et al. (2005) stated that root dry biomass of Populus species was reduced under mild and severe drought stress. In general, varieties having characters of deep and prolific root system were better able to sustain growth and yield under water-deficit conditions (Babu et al., 2001).

### Yield and Yield Related Attributes

Water deficit as well as sub-optimal temperatures reduce the growth and metabolic activities leading to reduction in agronomic and yield attributes of the crops. Rizza et al. (1994) reported that drought and chilling stress severely impaired the barley productivity. The chilling stress in chickpea at reproductive stage led to flower abortion and poor pod setting (Clarke and Siddique, 2004; Nayyar et al., 2005). In temperate growing areas, cold temperature is responsible for 30–40% reduction in rice yields because chilling stress resulted in degeneration of spikelets, panicle deformation and poor spikelet fertility (Andaya and Mackill, 2003). In grain crops, low temperature stress during reproductive development may induce flower abscission, ovule abortion, pollen sterility, pollen tube distortion, poor fruit set, and hence reduced final yields (Thakur et al., 2010). Whaley et al. (2004) found that chilling stress during stem elongation reduced the internode extension, caused spikelet death, and lowered biomass accumulation and grain yield in wheat. Low temperature at jointing and booting stages of wheat significantly decreased the number of productive tillers per plant (Li et al., 2015), and negatively affected the development of young spikes (Thakur et al., 2010).

Drought–induced decrease in yield and yield components of maize (Kamara et al., 2003), wheat (Barnabás et al., 2008), sugarcane (Vasantha et al., 2005), sunflower (Tahir et al., 2002), peanut (Furlan et al., 2012), and cotton (Pettigrew, 2004) has also been reported. In legumes, drought can dramatically reduce the seed yield by limiting flower and pod production, increasing flower and pod abortion, and reducing seed size (Davies et al., 1999; Fang et al., 2010). It has been estimated that drought stress may decrease the global chickpea production by 33% (Kashiwagi et al., 2015). In soybean, drought stress significantly reduced the number of branches and total seed yield (Frederick et al., 2001).

Conclusively, both drought and chilling temperature adversely affect yield and yield components of crop plants. Reductions in these traits could be the outcome of stress-induced modifications in metabolic and physiological activities and corresponding negative implication for plant reproductive organs with reduced productivity. Overall, onset of either drought or chilling is injurious to plants, nevertheless, plant reproductive stage is perhaps the most sensitive stage to these stress factors than pre- and post-reproductive stages.

## Physiological and Biochemical Responses

### Photosynthesis and Gaseous Exchange

Drought and chilling stresses alter the normal rates of photosynthesis and other gas exchange attributes in crop plants. While working on sugarcane, Sales et al., (2013) found that drought stress alone or in combination with chilling stress severely affected the leaf gas exchange, photochemical activity, CO_2_ assimilation, generation of energetic pressure at the PSII level, electron transport rate and transpiration. Under chilling stress, low photosynthetic rates in crop plants have been attributed owing to poor stomatal and mesophyll conductance of CO_2_, impaired chloroplastic development, restricted metabolite transport, decreased quantum efficiency and the quantum yield for CO_2_ assimilation (Nie et al., 1995; Haldimann, 1998; Sowiński et al., 2005). Jung and Steffen (1997) argued that chilling stress reduces photosynthetic efficiencies and enhances photo-inhibition process due to over excitation of thylakoid membranes and subsequent impairment of photosynthetic function. Moreover, chilling slows down the rate of CO_2_ fixation and restricts the NADP+ supplement available to accept electrons from the electron transport chain (Wise, 1995). On the other hand, drought stress reduces photosynthesis by inhibiting leaf area and decreasing the rate of photosynthesis per unit leaf area (Wahid and Rasul, 2005; Farooq et al., 2009b). Drought stress slows down carbon fixation rate by directly restricting metabolism or by limiting the CO_2_ entry into the leaf (Apel and Hirt, 2004; Farooq et al., 2009b). Imbalance between capturing and utilization of light (Foyer and Noctor, 2000), decrease in Rubisco activity (Bota et al., 2004), alterations in the photosynthetic pigments (Anjum et al., 2003), and damage to photosynthetic apparatus (Fu and Huang, 2001) are some of the major reasons for drought-induced reductions in photosynthesis. However, the drought-induced limitations of photosynthesis through metabolic distortions are more complex than stomatal limitations, and these mainly occur through reduced synthesis of photosynthetic pigments (Reddy et al., 2004). During drought stress, limited intercellular CO_2_ concentration leads to the accumulation of reduced photosynthetic electron transport components that can potentially reduce the molecular oxygen, resulting in the production of ROS, which are deleterious to photosynthetic apparatus (Basu et al., 2016). Although, chilling and drought stresses have some unique effects on photosynthetic functions of plants, yet both these stresses are detrimental for plant photosynthetic apparatus and light harvesting mechanism.

### Water Relations

Relative water content, leaf water potential, transpiration rate, stomatal conductance, and leaf and canopy temperature are the important attributes describing plant water relations (Farooq et al., 2009a). Sales et al. (2013) reported severe reductions in leaf water potential of sugarcane under the simultaneous occurrence of cold and drought stresses, compared with individual stress factor. Generally, the chilling-sensitive plants exhibit water stress symptoms under low temperatures, which are triggered by reduced root hydraulic conductivity followed by severe decline in leaf water potential and loss of turgor pressure (Aroca et al., 2003). McWilliam et al. (1982) and Lee et al. (1993) also reported that decline in root hydraulic conductivity and loss of stomatal control caused chilling-induced wilting in plants. Aroca et al. (2003) stated that low temperature-induced reduction in vapor pressure deficit between the leaf surface and the atmosphere decreased transpiration rate and root water uptake drastically. Chilling stress caused shoot water deficits in maize plants by significant reductions in root water uptake instead of leaf transpiration (Janowiak and Markowski, 1994). Likewise, Javier et al. (1997) reported that under chilling stress, root water uptake decreased in drought hardened plants similar to non-drought hardened plants, while transpiration rate was lower in drought hardened plants only.

Exposure of drought stress has also been known to substantially decrease the relative water content, turgor pressure, leaf water potential, and transpiration rate in a number of plant species (Siddique et al., 2001; Tezara et al., 2002; Nayyar and Gupta, 2006; Campos et al., 2011). Liu et al. (2004) reported that drought stress significantly declined the water potential of soybean roots, leaves, and pods, compared with control. Likewise, Reddy et al. (2004) observed that tissue water contents were decreased linearly with increase in drought severity. In short, chilling as well as drought stress cause disturbances in plant water relations; nevertheless such effects are likely to be more severe under the combination of both of these factors.

### Nutrient Relations

In addition to reduced plant growth and productivity (Tommasini et al., 2008), the nutrient uptake behavior of plants is also impaired under chilling (Xu and Huang, 2006) and drought stress (Hu et al., 2007). Temperature affects the physio-chemical and microbial processes in soils which may modify the plant-nutrient relationships (Yan et al., 2012). Poor root system of plants under chilling stress reduces the uptake of several nutrients, including N, P, and K (Pan et al., 1985; Steffens, 1986; Domisch et al., 2002; Yan et al., 2012). Under chilling stress, shoot growth of maize seedlings was reduced by the direct effect of low temperature on shoot meristems and by restricted supply of nutrients via roots (Hund et al., 2007). Farooq et al. (2009a) concluded that reduced root length, low hydraulic conductance, poor root branching, and thicker root axis under chilling stress lead to reduced mineral nutrient uptake in plants. However, the variations in such effects may arise based on plant species, stress period, physiological plant growth stage, or the frequency of changing the nutrient solutions.

Drought stress also decreases the availability, uptake, transfer and metabolism of nutrients (Farooq et al., 2009b). Exposure of drought stress in plants generally decreases both the uptake of nutrients by roots and translocation from roots to shoots (Hu and Schmidhalter, 2005). Drought-induced reductions in uptake and translocation of macro-nutrients (N, P, and K) have been reported in various plant species (Kuchenbuch et al., 1986; Subramanian et al., 2006; Asrar and Elhindi, 2011; Suriyagoda et al., 2014). Low soil moisture availability under drought stress reduced the root growth and the rate of nutrient inflow in terms of both per unit of root length and root biomass (Kuchenbuch et al., 1986). Moreover, drought stress causes the difference in active transport and membrane permeability of cations (K^+^, Ca^2+^, and Mg^2+^), thus resulting in decreased absorption of these cations via roots (Hu and Schmidhalter, 2005; Farooq et al., 2009b). Drought stress limits the activities of enzymes involved in nutrient assimilation. For instance, the nitrate reductase activities in the leaves and nodules of dhainicha (*Sesbania aculeata* L.) and common bean (*Phaseolus vulgaris* L.) were significantly decreased under drought stress (Ashraf and Iram, 2005). Apart from macro-nutrient, drought can induce the deficiencies of some micro nutrients, i.e., Mn, Fe, and Mo (Hu and Schmidhalter, 2005) whereas these micronutrients become increasingly available under well-watered conditions due to their conversion to more soluble and reduced forms (Havlin et al., 1999). In summary; there exists a significant interaction among the nutrient acquisition, soil moisture content and soil temperature. Both chilling and drought stresses decreases the availability, uptake, translocation and metabolism of nutrients in plants. Most of the previous reports have focused on the nutrient relation with individual stress, while rare studies have examined the nutrient acquisition in plants under combine stress factors. Therefore, quantification of the effects of combined chilling and drought stresses on the nutrient uptake, transport, and assimilation need to be investigated further.

### Oxidative Status

Imbalance between generation and safe detoxification of ROS represents metabolic state that is referred to as oxidative stress (Baier et al., 2005). Excess ROS accumulation causes protein oxidation, membrane lipid peroxidation, DNA and RNA damage, and may even lead to cell death (Mittler, 2002; Apel and Hirt, 2004). Hydrogen peroxide (H_2_O_2_), singlet oxygen (^1^O_2_), superoxide radicals (O^2-^), and hydroxyl radicals (OH^-^) are the major ROS produced in plants under unfavorable environmental conditions (Apel and Hirt, 2004). Generally, the production of ROS is linear with the severity of stress conditions.

Chilling and drought stresses cause oxidative damage by overproduction of ROS in plant cells (Xing and Rajashekar, 2001; Guo et al., 2006; Cruz de Carvalho, 2008; Farooq et al., 2009a,b; Gill and Tuteja, 2010; Shu et al., 2011). Chinnusamy et al. (2007) reported that chilling stress negatively affects the membrane properties and enzyme activities resulting in tissue necrosis. Under suboptimal growth temperatures, bundle sheath proteins were more sensitive to oxidative damage than those of mesophyll in maize leaves (Kingston-Smith and Foyer, 2000). Overproduction of ROS under chilling stress cause serious cellular damages by rapidly reacting with essential cellular structures (Sattler et al., 2000). Zhang et al. (1995) stated that balance between the formation and detoxification of ROS is crucial to cell survival under chilling stress. Likewise, numerous studies have reported the higher ROS accumulation and oxidative damage under drought stress (Mano, 2002; Blokhina et al., 2003; Cruz de Carvalho, 2008), owing primarily to limited carbon dioxide fixation and higher photorespiration (Cruz de Carvalho, 2008). Robinson and Bunce (2000) documented that considerably higher accumulation of ROS under drought stress resulted from the increased O_2_ photo reduction rate in chloroplasts.

To cope with oxidative damage the plants have evolved excellent antioxidative defense systems under unfavorable conditions like drought and chilling stresses. Increased activities/levels of several enzymatic (SOD, APX, POD, CAT, GR, GST, GPX etc.), and non-enzymatic (reduced and oxidized glutathione, ascorbic acid, α-tocopherol, and carotenoids) antioxidants have been reported in plants to maintain the cellular homeostasis and to mitigate the oxidative damage (Gill and Tuteja, 2010). The specific role of various enzymatic and non-enzymatic antioxidants in drought and chilling stress tolerance of various transgenic plants is summerized in **Table 1**. Chilling and drought stress tolerance of many crops is regulated by abscisic acid induced higher antioxidant enzyme activities (Lu et al., 2003; Zhou et al., 2005). In contrast, Thomashow (1999) claimed that chilling stress activated the antioxidant defense response by causing damage to membrane components and ROS-induced protein denaturation. Previously, it has been observed that greater activities/levels of antioxidants were well concominant with chilling tolerance in rice (Guo et al., 2006; Hussain et al., 2016), maize (Zhang et al., 1995; Prasad, 1997; Taka, 2004; Sharma et al., 2012), cucumber (Kang and Saltveit, 2002), and tabbaco (Deng et al., 2012). Likewise, several authors documented the role of different enzymatic and non-enzymatic antioxidants in drought tolerance of maize (Anjum et al., 2017), rice (Sharma and Dubey, 2005), wheat (Sairam and Saxena, 2000; Keles and Öncel, 2002), coffea (Lima et al., 2002), cotton (Ratnayaka et al., 2003), beans (Turkan et al., 2005), and alfalfa (Rubio et al., 2002). This implies that maintaining higher levels of antioxidants may contribute toward stress tolerance in plants.

**Table 1:** Role of enzymatic and non-enzymatic antioxidants for inducing drought and chilling tolerance in transgenic plants.

Gene	Source	Transgenic (host) Plant	Response in transgenic plants	Reference
Cu/Zn SOD	*Oryza sativa*	Tobacco	Drought stress tolerance	Badawi et al., 2004
Cu/Zn SOD	*Avicennia marina*	Rice	Drought stress tolerance	Prashanth et al., 2008
CAT	*Triticum aestivum*	Rice	Chilling stress tolerance	Matsumura et al., 2002
GST+CAT1	*Suaeda salsa*	Rice	Chilling stress tolerance	Zhao et al., 2006
GR+	*Arabidopsis thaliana*	Cotton	Chilling stress tolerance	Kornyeyev et al., 2003
GST	–	Rice	Chilling stress tolerance	Takesawa et al., 2002
Mn-SOD	*Nicotiana tabacum*	Alfalfa	Freezing and drought stress tolerance	McKersie et al. 1993, 1996
Proline P5CS	*Vigna aconitifolia*	Wheat	Drought stress tolerance	Vendruscolo et al., 2007
Proline P5CS	*Arabidopsis thaliana*	Petunia	Drought stress tolerance	Yamada et al., 2005
Proline P5CR	*Arabidopsis thaliana*	Soybean	Drought stress tolerance	Simon-Sarkadi et al., 2006
OsAPXa	*–*	Rice	Chilling stress tolerance	Sato et al., 2011
Capx	*Pisum sativum*	Tomato	Drought and chilling stress tolerance	Wang et al., 2005, 2006
APX3	*Arabidopsis thaliana*	Tobacco	Drought stress tolerance	Yan et al., 2003

### Osmotic Balance

Osmotic adjustment is the decrease of osmotic potential owing to the net accumulation of solutes in response to water deficit conditions (Zhang et al., 1999). Under osmotic stress, plants maintain the water relations by accumulating various osmotically active molecules/ions such as soluble sugars, organic acids, proline, glycinebetaine, potassium, calcium, and chloride ions. Osmoregulation/osmotic adjustment helps plants to sustain growth, photosynthetic rate, and assimilate partitioning under stressful conditions (Subbarao et al., 2000). Farooq et al. (2009a) stated that osmotic adjustment is directly related to the accumulation of osmolytes in different concentrations, which largely depends on stress severity, plant type and the growth stage at which the stress is imposed (Shao et al., 2006). As chilling stress has been reported to disturb plant osmotic balance and cause dehydration stress (Farooq et al., 2009a; Wang et al., 2016), therefore, under chilling and drought stress, osmotic adjustment assists the plants to maintain higher turgor potential (Zhang et al., 1999; Chaves et al., 2009). Morgan (1990) reported that osmotic adjustment is crucial in postponing the dehydration stress under water limited conditions. Greater accumulation of osmolytes is generally linked with drought tolerance (Blum, 2005) because these facilitate in maintenance of leaf turgor to improve conductance of stomata for efficient CO_2_ intake (Kiani et al., 2007), and promote the water uptake ability of roots (Chimenti et al., 2006). Mercado et al. (1997) reported that protein content in the tissues of chilling-sensitive plants are usually decreased with chilling stress, mainly because of reduced synthesis which, in turn, increases the level of free amino acids particularly proline (Jouve et al., 1993). Endogenous concentrations of proline were linked with chilling tolerance capability in maize (Songstad et al., 1990), and drought tolerance in rice (Hsu et al., 2003), and pea (Alexieva et al., 2001). Proline mainly accumulates in younger leaves and is recognized as the most improtant solute against abiotic stresses (Perez-Perez et al., 2009). Drought stress alters the endogenous levels of glycinebetaine. Cotton cultivars with higher endogenous glycinebetaine concentrations were better adapted to drought conditions than those which accumulaed less glycinebetaine (Naidu et al., 1998). Most of the studies have shown that osmotic potential of plants is more affected by drought as compared to chilling stress (Farooq et al., 2009b). However, further studies are needed to understand the alterations in plant osmotic balance under combined drought and chilling stresses.

## Molecular Responses

Crop growth and productivity is negatively affected by molecular alterations under various environmental stresses. Chilling and drought stress induce the expression of number of genes in plants, however, gene expression in response to both stresses are quite different (Liu et al., 1998). In Arabidopsis, expression of DREB1 (also called as CBFs or the c-box binding factors) and DREB2 families was affected in response to chilling and drought stresses, respectively (Stockinger et al., 1997; Liu et al., 1998). Likewise, Yamaguchi-Shinozaki and Shinozaki (1994) identified the dehydration-responsive element with the core sequence A/GCCGAC as a *cis*-acting promoter element, which were regulated in response to drought, and chilling stresses. Expression of homeodomain leucine zipper (4D-Zip) protein, which interacts with CaCBFIB (Mahajan and Tuteja, 2005) was highly regulated by drought and low temperature stresses (Kim et al., 2004). Chinnusamy et al. (2007) stated that cold temperature affects membrane fluidity, disrupts nucleic acid and protein structures, hinders water and nutrient uptake, and causes significant changes in the plant transcriptome. It also drastically disturbs the cellular metabolism either by directly decreasing the rates of biochemical reactions or indirectly by gene expression reprogramming. Chilling stress rapidly increased the levels of cytoplasmic calcium in Arabidopsis (Polisensky and Braam, 1996) and alfalfa (Monroy and Dhindsa, 1995) due to an influx of calcium from extracellular stores (Mahajan and Tuteja, 2005), suggesting that cell signaling and genes expression are altered under chilling stress. Lee and Lee (2003) observed that expression of majority of pollen-specific genes remained unaffected under chilling stress. However, Jouyban et al. (2013) reported that response of plants to chilling stress was associated with changes in gene transcription of low molecular weight proteins. Differences in genes expression in response to stress also occur among plant parts. Kreps et al. (2002) found that leaves and roots exhibited differential gene expressions during cold acclimation; and 86% of the cold-induced genes were not shared between leaves and roots. Chilling stress regulates the expression of ETHYLENE RESPONSE FACTOR/APETALA2 family transcription factors (e.g., CBFs), which may bind to *cis*-elements in the promoters of COR (cold-responsive) genes and induce their expression (Chinnusamy et al., 2007). Under cold stress, ICE1 (INDUCER OF CBF EXPRESSION1), was constitutively regulated in Arabidopsis, nevertheless, transgenic overexpression of ZAT12 down-regulated the expression of CBFs (Chinnusamy et al., 2007). Chilling stress also induces the accumulation of proline, which in turn induces the expression of genes having proline-responsive element (PRE and ACTCAT) in their promoters (Satoh et al., 2002; Chinnusamy et al., 2007).

Water deficit conditions also cause the fluctuations in gene expression (up- and down-regulation) in plants (Deng et al., 2009; Jain and Chattopadhyay, 2010). Drought stress altered the expression of dehydrin/late embryogenesis abundant (LEA) genes and molecular chaperones, which protect from protein denaturation in cell (Mahajan and Tuteja, 2005). Drought stress regulates the production of ABA in roots that is transported to the shoots leading to stomatal closure and restricted plant growth (Mittler and Blumwald, 2015). Drought-induced increase in ABA level regulates the expression of dehydrin genes (DHN 1/RAB and DHN 2), glycine rich protein gene, and a conserved element in proximal promoter region of gene RAB28 (Gomez et al., 1988; Pla et al., 1991; Campbell et al., 1998; Rorat, 2006). Under drought stress, miR398 was significantly up-regulated in peanut, while, it decreased the expression levels of isoforms Cu/Zn superoxide dismutase viz., AhCSD1-1/AhCSD1-2.1 and AhCSD2 (Park and Grabau, 2017). Bhargava and Sawant (2013) found that drought stress up-regulated the expression of genes involved in osmolyte synthesis, aquaporins, signaling molecules and transcription factors (TFs), and genes coding for LEA proteins. Moreover, TFs of ERF/AP2 family are known to bind with drought-response element present in promoters of many drought-response genes (Yamaguchi-Shinozaki and Shinozaki, 2005; Maruyama et al., 2009). Yang et al. (2010) observed that gene expression of TFs belonging to AP2/ERF, bZIP, HD-ZIP, bHLH, MYB, NF-Y, EAR, NAC, and ZPT2 families was regulated under drought stress. Bhargava and Sawant (2013) reported that drought stress induces the expression of a variant of histone H1 (called H1-S), which is involved in regulation of stress-responsive gene and plays a crucial role in stomatal closure (Scippa et al., 2004). Chinnusamy and Zhu (2009) reported that drought regulated the DNA demethylation in coding sequence of a glycerophosphodiesterase-like protein gene in tobacco, whereas, DNA hyper-methylation was regulated in pea under drought stress.

In summary, profound changes in gene expression occur under drought or/and chilling stresses (Maqbool et al., 2017). Many stress-inducible genes are responsive to both drought stress and chilling, while some genes are only regulated specifically by drought or chilling stress (Rabbani et al., 2003). Overall, majority of the transcription factors were up-regulated under these stresses, a few transcription factors having a role in primary growth processes were down-regulated (Bhargava and Sawant, 2013).

### Crosstalk Between Chilling and Drought Responses

Different abiotic stresses generally induce modify the gene expression in plants which can be stress-specific. Nevertheless, various points of crosstalk may occur amongst stress responses, and interplay of signaling pathways in plants. Cross talk refers to any instance of two pathways from different stress factors that converge and act in synchronization to alleviate the effects of stress (Knight and Knight, 2001). The plant responses to drought and chilling share various common domains including ROS and antioxidant response, calcium signaling, osmotic stress signal perception, transcription factors, hormones, dehydrins, and mitogen-activated protein kinase (MAPK) cascades, to name a few.

Identification of cross talk between different signaling cascades is important in strengthening our knowledge on plants responses to a specific stress condition. In recent era, researches dealing with the cross talk between different abiotic stress signaling pathways have paid attention on genes/gene products involved in two different stress conditions, and thus are known to be part of a shared response. The transgenic up- or down-regulation of these genes indicate that they play key role in enhancing tolerance to multiple abiotic stresses. Therefore, these genes can be crucial in conferring plant tolerance against combined stress factors often prevailing under field conditions.

Among four transcriptional regulatory systems in plants, two are ABA-dependent while two are ABA-independent (Shinozaki et al., 2003), and the crosstalk between these regulatory systems has been shown by molecular and genetic evidence in various reports. Genetic analyses suggested that there is no clear line of delineation between ABA-dependent and ABA-independent pathways, and the contributing components may often cross talk or even congregate in the signaling pathway (Kreps et al., 2002). Generally, the signaling pathways associated with chilling stress are reported to be rather less dependent on ABA than those involved in drought stress response (Shinozaki and Yamaguchi-Shinozaki, 2000; Shinozaki et al., 2003; Chen and Zhu, 2004; Zhang J.Z. et al., 2004). However, it is evident that some cross-talk, at least, does occur between different abiotic stress signaling pathways as the transcription of members of similar gene families is regulated and identical products are rather accumulated (Shinozaki et al., 2003; Chen and Zhu, 2004; Chinnusamy et al., 2004). For instance, the hydrophilic proteins (COR78 and COR15a) were accumulated in *Arabidopsis thaliana*, in response to both chilling and drought stress (Rajashekar, 2000).

Genomic analyses of stress-induced gene expressions using microarrays also have recently revealed crosstalk in stress-responsive genes (Kreps et al., 2002; Seki et al., 2002). Many drought-responsive genes were regulated by high-salinity stress or ABA; however, only 10% of drought-responsive genes were indeed cold-inducible as well. In fact, ABA is involved in regulation of osmotic stress-responsive genes but it does not specifically play a key role in low-temperature stress response (Kreps et al., 2002; Seki et al., 2002). Presence of ABRE (*cis* acting element) is generally important for ABA induced gene expressions (Seki et al., 2002).

As both drought and chilling result in cellular dehydration, it is not uncommon to expect some overlap in various signaling pathways focused on delivering protection against cellular dehydration. This commonality presumably occurs due to the involvement of the CBF regulon in the abiotic stress response (Fowler et al., 2005). Haarke et al. (2002) reported that CBF4/DREB1D expression was induced by the occurrence of osmotic-stress. The CBF/DREB1 family genes are mainly regulated by chilling stress, while CBF4 has been reported to be activated by drought stress which provides crosstalk between CBF/DREB1 and DREB2 regulatory systems. The drought-induced expression of CBF4 is controlled by ABA-dependent pathways, indicating that CBF4 may function in drought stress response that relies on ABA accumulation.

Calcium (a secondary messenger) and ROS network (which balances scavenging with production) are the ubiquitous components of both chilling and drought stress signaling pathways. Genes involved in Ca and ROS signaling constitute a crucial part of common molecular response of concurrent stresses in plants (Rabbani et al., 2003; Torres and Dangl, 2005). Generally, rapid increase in Ca levels in plant cells has been reported with ABA, drought, and chilling (Rabbani et al., 2003). Plants are also evolved in high degree of control over ROS toxicity, to extent level that ROS are exploited as signaling molecules (Timperio et al., 2008). The plant cell senses ROS through redox-sensitive transcription factors (e.g., heat-shock factors), which trigger functional proteins involved in the reinstatement of cellular homeostasis (Mittler et al., 2004).

Tommasini et al. (2008) investigated the drought- and low temperature-responsive genes in barley. About 44% genes specifically responded to drought, while only 3.8% were chilling specific, and 9.5% genes were shared between both stresses. Of 2,622 up-regulated genes, 13.0% were chilling specific, and 32.7% were drought specific suggesting that drought induced nearly three times as many stress specific changes as chilling (Kreps et al., 2002; Seki et al., 2002; Rabbani et al., 2003). Genes involved in osmotic stress signal perception (bZIP, MYB29, and MYB-CC), calcium signaling proteins, signal transduction (e.g., MAPK), protection of membranes and proteins (e.g., LEA proteins), and antioxidative defense mechanisms (e.g., glutathione related genes) were commonly regulated in chilling and drought stresses (**Table 2**). Chilling stress specifically activated the genes involved in cell cycle and DNA processing, signaling cascades and transcriptional control, protein modification and destination, oxidative stress, cellular transport, and cold shock response (**Table 2**). However, genes related to transcriptional regulation, protection of membranes and proteins, cell growth and cell cycle, water and ion uptake and transport, and ABA synthesis were specifically activated by drought stress (**Table 2**). Beck et al. (2007) concluded that accumulation of dehydrins is regulated under chilling, salinity, and ABA exposure along with drought stress. They further reported that extremely hydrophilic proteins as dehydrins not only protect biomembranes in ripening seeds (LEA proteins) but also accumulate in the tissue of drought-tolerant plants during cold adaptation.

**Table 2:** Shared and unique gene expression patterns in barley induced by low temperature and drought stresses.

General process	Detailed process	Annotation	Functions
**Genes responsive to low temperature and drought**			

Transcriptional control (osmotic stress signal perception)	–	bZIP	Basic leucine zipper (bZIP) proteins, are ABA responsive transcription factors active under osmotic stress and are involved in regulation of transcription
		MYB29	Encodes RVE6 and RVE8, which are involved in regulation of circadian rhythm
		MYB-CC	Myb-like HTH transcriptional regulator family protein are involved in regulation of transcription.
Signal transduction	Protein kinases	MAPK	Mitogen-activated protein kinase are involved in processes of differentiation and transcription regulation
Protection of membranes and proteins	LEA proteins	Lea14-A	Late embryogenesis abundant proteins are involved in embryo development
	Praline	Delta1-pyrroline-5-carboxylate dehydrogenase	These proteins are involved in oxidation-reduction process and proline metabolic process
		Disulfide oxidoreductase	
	Galactinol	Galactinol synthase	Galactinol synthase is involved in galactose metabolic process
Oxidative stress response (Rescue and defense mechanism)	Glutathione	ATGR1;glutathione-disulfide reductase	These genes are involved in the synthesis of antioxidants


	Methyltransferase	Methyltransferase	
	Aldehyde dehydrogenase		
Ion and Water uptake and transport		Na^+^/H^+^ antiporter	Encodes a vacuolar sodium/proton antiporter involved in ion homeostasis

**Genes responsive exclusively to low temperature**			

Embryonal development	LEA proteins	LEA protein	LEA proteins are involved in membrane stabilization and inhibition of ice crystallization


		LEA group 1 domain-containing proteins	
Water and ion uptake and transport		Calmodulin 6 (CAM6)	CALMODULIN 6 is involved in calcium-mediated signaling
Cell rescue and defense	Cold shock response	ELIP1	EARLY LIGHT-INDUCABLE PROTEINS are involved in regulation of chlorophyll biosynthetic process and transmembrane transport
		OEP16 (outer envelope protein 16)	Encodes AtOEP16 involved in protein import into mitochondrial matrix
	Oxygen radical detoxification	NTRA (NADPH-dependent thioredoxin reductase 2)	NADPH-dependent thioredoxin reductase is involved in cell redox homeostasis and oxidation-reduction process

**Genes responsive exclusively to drought**			

Signaling and transcriptional control		bZIP	Basic leucine zipper (bZIP) proteins are ABA responsive transcription factors active under osmotic stress
		AP2/DREB2A/DRF1	DEHYDRATION-RESPONSIVE ELEMENT BINDING PROTEIN 2 family is involved in heat acclimation and positive regulation of transcription
		HD-ZIP	HD-ZIP is related to genes responsive to dehydration and involved in cell differentiation
		ERF1	Eukaryotic Release Factor is involved in regulation of growth, translational termination
Protection of membranes and proteins	Sugars	Sucrose phosphate synthase 1F	These molecules accumulate in the cytoplasm at high concentrations under osmotic stress without interfering with normal metabolism and may have a primary role in maintaining turgor, cell structures stabilizing proteins and scavenging ROS


		betaine aldehyde dehydrogenase	
		ATP-binding galactokinase	
		Sucrose synthase 2	
	LEA proteins	LEA2	LEAs are typically induced by ABA or osmotic stress and are involved in oxidation-reduction process


		LEA3	
	Cell rescue and defense mechanisms	Peroxiredoxins	Thioredoxin superfamily proteins are involved in cell redox homeostasis and oxidation-reduction process
		Cu–Zn superoxide dismutase	These genes are involved in both a CCS-dependent and -independent pathway
		HSP91	Heat shock protein genes are induced by drought stress, and contribute in cell rescue and defense mechanisms
		HSP70	
ABA biosynthesis	–	9-*cis*-Epoxycarotenoid dioxygenase 2	Drought induced genes are involved in the synthesis of ABA and play important role in drought stress adaptation
Water and ion uptake and transport	–	CorA like Mg^2+^ transporter	Involved in ion uptake and cell growth

*Data regarding gene expression is taken from the study of Tommasini et al. (2008).*

Each stress is a multigenic trait and, thus, the manipulation of such genes may lead to alteration of a large number of genes as well as gene products. A deep insight into transcription factors regulating these genes, the products of the major stress-evoked genes, and cross talk between different pathways and responses should remain an area of future research activities. The fact that these cross talk genes regulate both chilling and drought response of plants highlights their importance in enhancing plant tolerance to combined stress factors. However, this should be validated by conducting studies on concurrent stresses wherein expression of cross talk genes needs to be examined.

## Management Strategies

Both drought and chilling stresses have adverse effects on the growth and productivity of crop plants (Liu et al., 1998), and plant tolerance to these stresses might have certain protective mechanisms in shared (Stockinger et al., 1997). Plants often develop a cascade of strategies through physiological, biochemical, and molecular modifications at cell level to cope with these stresses. Studies have shown that mechanisms of plant tolerance to chilling and drought stresses can be regulated through developing tolerant plant genotypes, genetic modifications, seed treatments, application of plant growth regulators and compatible solutes and use of plant mineral nutrients.

### Selection and Breeding Strategies

Integration of conventional, molecular and omic-based techniques can successfully be employed to develop tolerant plant genotypes (Maqbool et al., 2017). Conventional breeding has been instrumental in developing chilling tolerant cultivars in various crops (Jha et al., 2017). Various rice varieties, e.g., ‘Koshihikari’ in Japan, ‘Silewah’ in Indonesia and ‘Padi Labou Alumbis’ in Malaysia were released for chilling stress tolerance by conventional breeding method (Ahmad P. et al., 2014). Based on both open air and controlled conditions, Rodriguez et al. (2007) reported that ‘EP80 × Puenteareas’ population in maize was an important source of low temperature tolerance. Implementation of the modern omics approaches and identification of QTLs/genes for chilling tolerance can significantly support crop improvement strategies aimed to develop high yielding cultivars under low-temperature condition (Ahmad P. et al., 2014; Jha et al., 2017). In rice, selection using different parameters led to the development of low temperature tolerant genotypes such as ‘HSC55,’ ‘M103,’ and ‘Jyoudeki’ based on low spikelet sterility (Farrell et al., 2006; Ye et al., 2009). Suh et al. (2010) reported that phenotyping selection and SSR makers’ identification methods are helpful in screening of plant genotypes against chilling stress. They found three QTLs responsible for seed setting percentage under chilling stress by using a recombinant inbred line (RIL) population developed by tropical japonica × temperate japonica.

Under drought stress conditions, estimating final grain yield is a simple and easy method for screening tolerant crops. Promising tolerant genotypes can be identified by direct selection of grain yield under water deficit conditions (Verulkar et al., 2010). Hence, conventional breeding approach includes the selection of crop plants for its yield and related attributes under drought conditions (Ahmad P. et al., 2014). In general, breeding strategies have been employed to manipulate the genetic makeup of crops for enhancing their tolerance against drought (Maqbool et al., 2017), however, marker assisted selection (MAS) proved better than classical breeding for screening traits associated with drought tolerance (Ahmad P. et al., 2014) and enhancing drought tolerance in plants (Jongdee et al., 2002). The molecular basis of drought tolerance is generally based on QTL analysis and segregation mapping (Ali et al., 2017). The identification of QTLs associated with drought tolerance is an important tool for MAS of plants with desired characters (Farooq et al., 2009b). Marker assisted recurrent selection involves the accumulation of more superior alleles responsible for drought tolerance in plants (Varshney et al., 2012). A large number of QTLs have already been identified for several drought tolerance traits, nevertheless, epiQTLs and Epistatic QTLs discovered in future can be used for molecular breeding (Gupta et al., 2017). Sequential or simultaneous transfer of QTLs at different developmental stages of rice may lead to cultivars with increased drought tolerance capacities (Lang et al., 2013). Overexpression of an LEA gene has been reported to increase drought tolerance in rice (Xiao et al., 2007). Furthermore, while discussing the constraints in breeding approaches, Ouk et al. (2006) reported that low heritability of grain yield and lack of effective selection criteria are the main hindrances to develop drought tolerant cultivars. Similarly, Kumar et al. (2008) reported that many previous trials on drought stress tolerance failed to impose severe stress, therefore true and precise drought-tolerant lines were not selected. Although, conventional breeding methods for drought tolerance have been used for a long time, nevertheless, advancements in genomics and molecular breeding approaches would have the significant role in the development of drought tolerant cultivars (Kumar et al., 2015). Moreover, selection of crop cultivars for their unique responses to drought and chilling stress might be helpful for breeding programs to develop stress tolerant plant types.

### Molecular and Functional Genomics Approaches

Plants respond to drought or chilling stress through a series of mechanistic changes in morpho-physiological features (Shinozaki and Yamaguchi-Shinozaki, 1996). Many genes that respond to drought or low temperature at the transcriptional level have been previously identified (Skriver and Mundy, 1990; Thomashow, 1994; Kasuga et al., 2004; Yadav, 2010; Todaka et al., 2015; Ali et al., 2017; Kaur and Asthir, 2017). However, the products of some specific stress-responsive genes could have significant roles in mitigating stress-induced damage through still elusive mechanisms (Shinozaki et al., 2003). Liu et al. (1998) stated that ABA is generally produced under both drought and chilling stresses and has significant contributions in stress tolerance in plants. They further stated that overexpression of various regulatory elements induces drought and low temperature tolerance in plants (Liu et al., 1998). Kasuga et al. (2004) reported that overexpression of DREB1A enhanced drought and chilling tolerance in tobacco plants. Furthermore, genes encoding transcription factors were recognized better for improving stress tolerance in plants than various other genes responsible for cold tolerance in Arabidopsis and rice (Yadav, 2010). Tuberosa et al. (2008) stated that identification of QTLs involved in chilling stress tolerance may directly relate to acquire valuable genetic diversity in the physiological features in plants. Low-temperature restrictions have been overwhelmed by the support of cold-tolerant genes in genetically modified crops (Sanghera et al., 2011). Chilling stress induces three C-repeat binding factor genes or dehydration-responsive elements such as DREB1B (AtCBF1), DREB1C (AtCBF2), and DREB1A (AtCBF3) in Arabidopsis (Yadav, 2010). Cold tolerance in tobacco can also be enhanced by the transgenic overexpression of the chloroplast omega-3 fatty acid desaturase gene (Kodama et al., 1994). Upchurch (2008) also reported the involvement of omega-3 desaturases to establish chilling tolerance in plants. Liu et al. (2008) described that the overexpression of Lefad7 in transgenic tomato could enhance the chilling stress tolerance. Lukatkin et al. (2012) reported that plant response to low temperature is linked with the extent of change of gene transcription of low molecular weight proteins. Mahajan and Tuteja (2005) described that CBF binds with COR genes carrying CRT/DRE elements, and the over-expression of these COR genes have an imperative role for chilling tolerance and cold acclimation in plants (Thomashow, 1994; Stockinger et al., 1997; Yadav, 2010). These findings signify the roles of cold-inducible genes to protect plant cells against cold stress (Yadav, 2010).

Mechanism of drought tolerance is related to the drought induced regulations of multiple genes expression (Lang and Bui, 2008). Drought tolerant plants developed by recent genomics approaches require clear understandings of the genetic basis of drought tolerance (Xiong et al., 2006), and identification of transcriptions factors associated with drought tolerance is important in this regard. Recently, an active form of DREB2 was found to transactivate target stress-evoked genes that led to better drought stress tolerance in Arabidopsis (Todaka et al., 2015). Ali et al. (2017) also reported the involvement of water stress induced transcription factors (DREB2A and DREB2B) in the expression of various genes responsible for drought stress tolerance in plants. Kaur and Asthir (2017) reported that responses of ABA-responsive elements-binding proteins (AREB) at both transcriptional and post-transcriptional levels of drought also determine the drought tolerance abilities of the plants. Moreover, Afzal et al. (2016) described the importance of aquaporins in regulating the plant-water relations and could be promising targets for developing drought tolerant plant genotypes. Differential responses of two contrastive barley genotypes were found in low-molecular dehydrins under water deficit conditions which were involved in drought stress tolerance (Škodáèek and Prášil, 2011). Bruce et al. (2002) enlighten the necessities to advent the whole genomics to identify the key genes involved in the regulations of drought stress tolerance in plants. Since, drought tolerance is a genetically controlled phenomenon, therefore, identification of QTLs for membrane stability and other functionally related phenomenal genes needs to be explored in future by using bio-informatics whilst many of them has already been characterized (Tripathy et al., 2000; Fu et al., 2007).

Some studies have been shown that some genes expressed by both drought and cold stress concurrently whilst some are only responsive to individual stress (Thomashow, 1994; Liu et al., 1998; Kasuga et al., 2004; Sanghera et al., 2011). There have been several efforts in improving tolerance toward combine stress factors such as concurrent chilling and drought stresses. The improved tolerance to chilling and water deficit stress in Arabidopsis was attained by overexpression of CBF4 (Haake et al., 2002; Sanghera et al., 2011). In addition, the overexpression of DREB1A has been found to have linked with drought and chilling stress in wheat, groundnut and tobacco plants (Kasuga et al., 2004; Pellegrineschi et al., 2004). In order to improve tolerance against chilling and drought stress, applications of various molecular and engineering strategies are needed. It is well evident that the integration of traditional and molecular breeding approaches with genetic engineering and MAS may help the scientists to improve individual and concurrent environmental stresses in crop plants (Chaves and Oliveira, 2004).

### Agronomic and Physiological Measures

Plants respond to drought and chilling stress via series of agronomic and physiological processes. Exogenous application of compatible solutes, plant growth hormones, plant mineral nutrient and seed priming can be effective in alleviating the adverse effects of drought and chilling stresses.

#### Application of Compatible Solutes

Compatible solutes protect the plants from osmotic stress by not posing any detrimental effects on enzymes, membranes, and other macromolecules even at higher concentrations (Cechin et al., 2006; Kiani et al., 2007). Compatible solutes include glycinebetaine, soluble sugars, proline, sugar alcohols, trehalose and some organic acids (Kiani et al., 2007). Many of the osmolytes are organic solutes in nature whilst some of the essential ions such as K^+^ (Yokoi et al., 2002).

Amongst various osmolytes, proline has been found to have multiple roles in plant stress regulation and tolerance potential (Yamada et al., 2005; Farooq et al., 2009a,b). Proline and polyamines have been reported to impart chilling tolerance in maize (Songstad et al., 1990). Proline retains the potential to relieve low temperature injury in cold-sensitive plants. Although, most of the chilling sensitive plants also accumulate proline but the levels of accumualation are not enough to induce cold tolerance, therefore, exogenous application of proline can be a potential option to enhance its level in plants. The beneficial role of proline in augmenting drought tolerance in rice (Hsu et al., 2003) and pea cultivars (Alexieva et al., 2001) has been well reported.

Soluble sugars and sugar alcohols serve as osomoregulators, signelling molecules, and cryoprotectants in plants (Ruelland et al., 2009) and helps in cleansing stress-generated ROS (Van den Ende and Valluru, 2009). Chilling tolerance has been observed by increase of total soluble sugars in many plant species (Levitt, 1980; Farooq et al., 2009a; Wang et al., 2016). Soluble sugars reduce osmotic potential and enhance water absorption under water limited conditions, and thus improve stress tolerance in plants (Farooq et al., 2009b).

Exogenous application of glycinebetaine play critical roles in abiotic stress tolerance in plants by modifying antioxidant activities, membrance integrity, osmotic adjustment and ROS detoxification (Somersalo et al., 1996; Xing and Rajashekar, 2001; Farooq et al., 2008). Xing and Rajashekar (2001) reported that glycinebetaine accumulation was positively related to the cold stress tolerance in plants. Glycinebetaine improved the growth of *Solanum tuberosum* lants under low temperature conditions (Somersalo et al., 1996) and freezing tolerance in *A. thaliana* (Xing and Rajashekar, 2001). Its foliar applicationwas helpful in drought tolerance of several plant species (Xing and Rajashekar, 2001; Farooq et al., 2009b). In short, application of campatible solutes such as glycinebetaine, soluble sugars, proline, and polyamines can be helpful in imparting drought and chilling tolerance in crop plants.

#### Application of Plant Growth Regulators

Plant growth hormones have a dynamic role for increasing the ability of plants to acclimatize against abiotic stress conditions. Auxins (IAA), gibberellins (GA_3_), cytokinins (Cks), ethylene, abscisic acid (ABA), salicylic acid (SA), jasmonates (JA), and brassinosteroids (BRs) have been widely studied in plants under different abiotic stresses. These plant hormones are being used efficiently under stress conditions for enhancing crop production. Plants normaly synthesize various types of phytohormones under individual and concurrent abiotic stresses depending upon the strength of their defense mechanism (Nadeem et al., 2016). ABA is synthesized in response to both drought and chilling and contribute in stress tolerance of crop plants (Liu et al., 1998). ABA, JA, and SA often activate phosphoprotein cascade pathways that led to expression of genes associated with cold stress tolerance in plants (Kolaksazov et al., 2013). Greater chilling tolerance has been recorded in ABA-treated maize (Xin and Li, 1992), and levels of ABA are increased in chilling tolerant genotypes (Farooq et al., 2009a). Under drought stress, ABA plays a key role in plant signaling betweeen shoot and root, that results in water-saving antitranspirant response, notably stomatal closure and reduced leaf expansion (Wilkinson et al., 2012). ABA is also involved in robust root growth and other morphological modifications under drought stress (Giuliani et al., 2005). During drought stress, increased accumulation of ABA decreases the Cks contents while triggers the ABA/Cks ratio in plants (Wani et al., 2016). Under water stress conditions, plant growth regulator including BRs and ABA significantly improved chlorophyll content and increased water potential in soybean (Zhang M. et al., 2004). Khan et al. (2015) reported that application of BRs led to considerable improvements in plant tolerance against chilling stress in terms of plant growth, photosynthesis and antioxidant system in tomato plants. Reguera et al. (2013) stated that drought tolerance in rice was concomitant with the accumulation of Cks. Drought tolerance induced by Cks has also been observed in tobacco (Rivero et al., 2007) and wheat (Shang, 2000). Kaya et al. (2006) reported that drought tolerance can also be enhanced by the application of GA_3_ in maize. Exogenous GA_3_ application breaks the rosette by rapid enlargement of differentiated tissues under cold stress (Khan et al., 2017). Similarly, JA has been reported to trigger the plant defense responses to abiotic stresses including drought and chilling (Pauwels et al., 2009; Seo et al., 2011). Exogenous application of methyl jasmonate improved chilling tolerance in wheat by modulating antioxidant defense system and production of soluble protein content (Qi et al., 2005). The relationship between enhanced ethylene levels and chilling tolerance in crop plants has also been reported by Chu and Lee (1989) and Zhao et al. (2014).

Salicylic acid is also considered as poterntial chemical compound for drought and chilling tolerance in several plant species (Farooq et al., 2009a,b). SA application was found to be effective in alleviating the chilling (Miura and Tada, 2014) and freezing injury (Tasgin et al., 2003). In maize, the harmful effects of low-temperature stress were decreased by the application of SA (Janda et al., 1999; Wang et al., 2012). Low concentrations of applied SA have also been reported to improve drought tolerance in crop plants (Miura and Tada, 2014). Horváth et al. (2007) reported that significant improvement in performance of winter wheat was related with SA-induced higher activities of CAT under drought stress. Exogenous auxin application improved wheat yield significantly under drought stress conditions (Abdoli et al., 2013), and overepxression of auxin efflux carrier gene (OsPIN3t) in rice was linked with enhanced drought tolerance (Zhang et al., 2012).

Non-hormonal growth regulators, e.g., triazoles, paklobutrazol, chlorocholinchloride, meflidid, and unikonazol (Lurie et al., 1994; Feng et al., 2003) are also used to enhance drought and chilling tolerance in crop plants (Feng et al., 2003; Lukatkin et al., 2012). Still and Pill (2003) observed that foliar applications of paclobutrazol remained effective in improving drought tolerance in tomato seedlings. These evidences suggest the exogeneous application of various plant growth regulators is a potent method to improve the drought and chilling tolerance in plants. However, future research should focus on developing genetically engineered or transgenic plants that have the ability to produce specific hormones by using biotechnological and molecular techniques. These transgenic plants would be capable to grow sucessfully under stressful environments.

#### Use of Mineral Nutrients

Mineral nutrients play important role in improving plant tolerance against stress conditions (Marschner, 1995; Waraich et al., 2012). To alleviate the adverse effects of abiotic stresses, nitrogen (N) fertilization has been reported to have significant role in stress alleviation (Waraich et al., 2011a). Nitrogen as nitric oxide (NO) is a highly reactive that protects plant against stress conditions by acting as scavenger to ROS (Wendehenne et al., 2001; Waraich et al., 2012). Under drought stress, phosphorus (P) helps plants to maitain leaf water potential which, in turn, enhances stomatal conductivity and photosynthetic rates (Waraich et al., 2011b). Supplementation of K doses and its uptake in plant parts could be beneficial for obtaining reasonable yields under drought conditions (Valadabadi and Farahani, 2010). K-induced increases in proline and free sugar contents in rice were reported by Pandey et al. (2004) under water deficit conditions. Kafkafi (1990) reported that low temperature injuries can be avoided in carnation plants by supplimenting K in irrigation water.

Calcium has been reported enhance the concentrations of amino acids, polyamines (putrescine and spermidine) and chlorophyll content in red spruce under low temperature stress (Schaberg et al., 2011). Waraich et al. (2012) reported that Ca is an essential nutrient for stomatal closure in chilling tolerant genotypes. Application of Ca in *Vicia faba* enhanced plant biomass, plant water relations, and chlorophyll content while reduced membrane leakage (Abdel-Basset, 1998). Magnesium (Mg) also improve root morphological charcaters which helps to increase uptake of water and nutrients via roots, whlist boron nutrition enhances sugar transport in the plant body and improves seed germination and grain formation process (Waraich et al., 2012). Application of Se was found to improve plant tolerance against chilling (Hawrylak-Nowak et al., 2010) and drought stress (Valadabadi et al., 2010). Silicon application improved the plant water status and biomass accumulation in sorghum (Hattori et al., 2005) and wheat (Gong et al., 2003) under drought stress. These studies suggest that use of plant mineral nutrient is a potiential option to achieve better crop growth and productivity and to alleviate the dterimental effects of drought and/or chilling stresses in a sustainable way.

#### Seed Priming

Seed priming is considered to improve the abiotic stress tolerance (including chilling and drought) in different plant species (Hasanuzzaman and Fujita, 2011; Hussain et al., 2016; Wang et al., 2016; Samota et al., 2017). Seed priming promotes the germination related metabolic functions without radicle protrusion from seeds (Hussain et al., 2016; Wang et al., 2016; Zheng et al., 2016). It is a useful technique to acclimatize against stress conditions (Samota et al., 2017) by modulating physio-biochemical processes in developing seedlings (Hasanuzzaman and Fujita, 2011). Seed- priming with plant hormones and other chemical compounds can play effective role in alleviating the adverse effects of drought and chilling stresses at earlier growth stages. Seed priming activated antioxidant defense system thus improved seed germination and early growth in tobacco seedlings (Xu et al., 2011). Seed priming with ascorbic acid, SA and H_2_O_2_ improved seedling establishment under suboptimal temperature (Ahmad I. et al., 2014). Hussain et al. (2016) found that the seed priming with selenium and SA were effective in improving chilling tolerance in rice cultivars. Likewise, seed priming either with 2.5% K_2_HPO_4_ or 2.5% K_2_HPO_4_ + KNO_3_ were observed to induce chilling tolerance at early growth stages than non-primed seeds (Farooq et al., 2009a). Seed priming with SA improved relative water and chlorophyll contents, antioxidant activities, plant biomass and grain yield of wheat (Singh and Usha, 2003), whereas selenium priming regulated activities/levels of enzymatic and non-enzymatic antioxidants in rapeseed under drought stress (Hasanuzzaman and Fujita, 2011). The priming of tomato and bean seeds with 0.1–0.5 mM SA improved plant tolerance to drought and low temperature stresses (Senaratna et al., 2000). Significant improvements in physio-biochemical features and the expression of rice drought-responsive RD1 and RD2 genes of AP2/ERF family in drought tolerant and sensititve rice genotypes were observed when seeds were primed with methyl jasmonate, SA, and paclobutrazol (Samota et al., 2017). Above evidence suggest that seed priming offers a realistic solution to increase the stress tolerance of plants against the drought and suboptimal temperatures.

## Conclusions and Future Perspectives

**FIGURE 1:**
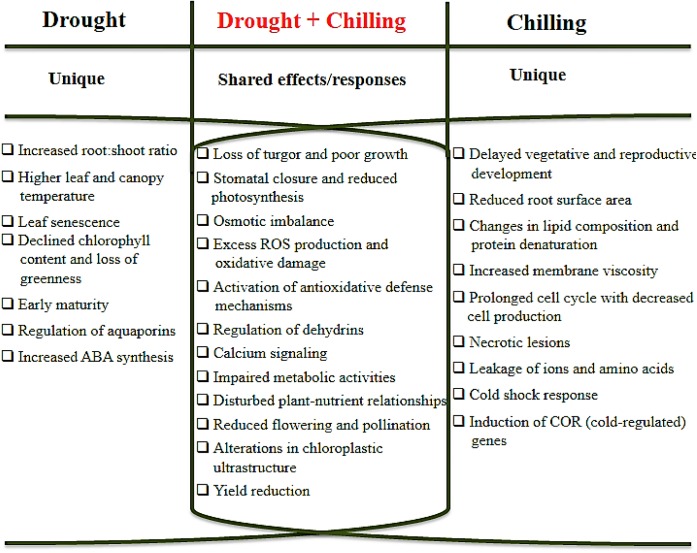
A schematic diagram of unique and shared effects of drought and chilling stresses on crop plants.

Emergence of intricate stress combinations and their impacts on crop growth and productivity in modern day agriculture are the outcomes of global climate change. Climate change is a multi-facet field that could have long-term impacts in the form of different abiotic stresses. Both drought and chilling stresses are amongst those abiotic stresses that have deleterious effects on crop plants. Plants depict a wide range of responses to these stresses (**Figure 1**) that could lead to significant reductions in crop yields or some time complete crop failure. Actually co-occurrence of drought and chilling stresses is a major challenge for crop production under field conditions instead of individual stress. Both stresses are also characterized by unique changes in plant growth and development starting from seed germination to yield. The ability of plants to withstand these stresses also differs from species to species. Above evidences affirms that plant responses to combined stresses are unique than those to individual stress factor. Being unique, chilling stress can change the root morphology more adversely than drought stress. In contrast, reduced crop yields as a result of poor dry matter accumulation, reduced flower and pod formation, increased flower and pod abortion, and small seed size are the outcomes of both stresses. Other noticeable effects are diminished nutrient uptake behavior, CO_2_ diffusion into chloroplast and photosynthetic system in plants. In general, interaction between drought and chilling conditions have found to be additive for almost all plant growth and physiological attributes leading to enhanced damage under combine stresses. In order to overcome the effects of chilling and drought, plants induce biochemical, physiological and molecular modifications that improve tolerance against these stresses. Concurrent occurrence of chilling and drought cause the osmotic as well as oxidative stress on shared basis. Plants counter-balance these adversities by accumulation of compatible solutes and ROS detoxifying proteins, and regulating the activities of antioxidant enzymes. Gene expression is also changed in response to both drought and chilling stresses; some of these genes are regulated only by drought and some genes are chilling-inducible only. To date, various novel approaches have been tested in minimizing the negative effects of combine stresses. Despite of the main improvements there is still a big room for improvement in combination of drought and chilling tolerance. For proper understanding of the plants responses to drought and chilling stress, the experiments should be designed under field conditions. Furthermore, future studies should focus on developing genetically engineered plants by using molecular and biotechnological approaches that have the capability to create a particular response against drought and chilling stress. Transcriptomics and proteomics analysis, genomic sequencing and bioinformatics can help to determine the common and unique genes modulated under stress condition. Thus, these techniques can improve the sophisticated and efficient network in plant response to drought and chilling stresses and subsequently help in the improvement of plant tolerance and productivity.

## Author Contributions

HH, SH, and LW conceived the idea of the review and prepared the initial outline. HH, SH, UA, SA, and SM gathered the literature and contributed to writing the different sections. AK and LW provided the technical guidance and editing support.

## Conflict of Interest Statement

The authors declare that the research was conducted in the absence of any commercial or financial relationships that could be construed as a potential conflict of interest.
